# Role of apparent diffusion coefficients with diffusion-weighted magnetic resonance imaging in differentiating between benign and malignant bone tumors

**DOI:** 10.1186/1477-7819-12-365

**Published:** 2014-11-29

**Authors:** Tingting Wang, Xiangru Wu, Yanfen Cui, Caiting Chu, Gang Ren, Wenhua Li

**Affiliations:** Department of Radiology, Xinhua Hospital affiliated to Shanghai Jiao Tong University School of Medicine, 1665 Kong Jiang Road, Shanghai, 200092 China; Department of Pathology, Xinhua Hospital affiliated to Shanghai Jiao Tong University School of Medicine, 1665 Kong Jiang Road, Shanghai, 200092 China

**Keywords:** Apparent diffusion coefficient, Bone tumors, Differentiation, Diffusion-weighted imaging

## Abstract

**Background:**

Benign and malignant bone tumors can present similar imaging features. This study aims to evaluate the significance of apparent diffusion coefficients (ADC) in differentiating between benign and malignant bone tumors.

**Methods:**

A total of 187 patients with 198 bone masses underwent diffusion-weighted (DW) magnetic resonance (MR) imaging. The ADC values in the solid components of the bone masses were assessed. Statistical differences between the mean ADC values in the different tumor types were determined by Student’s *t*-test.

**Results:**

Histological analysis showed that 84/198 (42.4%) of the bone masses were benign and 114/198 (57.6%) were malignant. There was a significant difference between the mean ADC values in the benign and malignant bone lesions (*P* <0.05). However, no significant difference was found in the mean ADC value between non-ossifying fibromas, osteofibrous dysplasia, and malignant bone tumors. When an ADC cutoff value ≥1.10 × 10^−3^ mm^2^/s was applied, malignant bone lesions were excluded with a sensitivity of 89.7%, a specificity of 84.5%, a positive predictive value of 82.6%, and a negative predictive value of 95.3%.

**Conclusions:**

The combination of DW imaging with ADC quantification and T2-weighted signal characteristics of the solid components in lesions can facilitate differentiation between benign and malignant bone tumors.

## Background

Preoperative characterization of benign, malignant, and tumor-like bone lesions is important in order to make informed choices regarding treatment strategies. Although plain film radiography is still considered the first-line imaging modality for assessing the nature and defining the characteristics of primary bone lesions, certain areas of the musculoskeletal system may be difficult to profile in plain films due to overlapping structures [1]. Computerized tomography can provide more detailed information, including focal destruction, periosteal reaction, subtle matrix mineralization, and endosteal scalloping [2]. Magnetic resonance (MR) imaging is considered the most advanced imaging technique and the most sensitive for evaluating changes in bone-marrow and defining the extent of a lesion, particularly when plain films or computerized tomography findings are suboptimal or indeterminate; however, it is not always the most specific. Despite their respective advantages, none of these imaging techniques can reliably differentiate between benign and malignant bone tumors, as many lesions are non-specific and display varying imaging characteristics on T1- and T2-weighted images. MR characterization of bone lesions can be improved by the use of MR diffusion-weighted (DW) imaging, as this is sensitive to changes in the microdiffusion of water into both intracellular and extracellular spaces. The advantage of evaluating diffusion is the ability to probe the cellularity of neoplasms. Apparent diffusion coefficients (ADC) are largely proportional to the ratio of extracellular and intracellular components, cell density, intracellular organelles, matrix fibers, and soluble macromolecules. Tumors with different levels of cellularity have different ADC values corresponding to changes in restricted diffusion [3–6].

The purposes of this study were to clarify the relationship between ADC values in the solid components of bone masses and to evaluate its supplementary use in differentiating between benign and malignant bone tumors.

## Methods

### Patient selection

This study received approval from our institutional (Xinhua Hospital) review board and the requirement to obtain written informed consent was waived. A total of 178 patients who had been diagnosed with bone tumors between January 2005 and March 2014 were enrolled in this study. The selection criteria were as follows: the diagnosis was confirmed by histological biopsy or surgery, MR imaging was performed using a 3.0 T magnet, and both conventional MR imaging with DW imaging and contrasted-enhancement MR imaging were performed. A retrospective evaluation of the MR imaging data was undertaken.

#### MR imaging protocol

All patients underwent MR imaging with a 3.0T MR unit (GE Medical Systems; Milwaukee, WI, USA). Axial non-contrast T1-weighted (TR/TE, 400 to 500/10 to 12 ms) and axial T2-weighted (TR/TE, 4,000 to 5,000/100 to 120 ms) imaging were performed with chemical shift-selective fat saturation pulse using the following parameters: slice thickness, 5 mm; gap, 1 mm; field of view, 20 to 40 cm; matrix, 256 × 256; and excitation, 2. Sagittal T1-weighted and T2-weighted (TR/TE, 3,000 to 5,000/100 to 110 ms) fast spin-echo imaging without chemical shift-selective fat saturation pulse were also performed using the parameters described above. DW-MR imaging was performed in the axial or sagittal plane prior to administration of contrast medium using a single-shot echo-planar imaging sequence (TR/TE effective range, 6,000 to 8,000/70 to 100 ms; slice thickness/intersection gap, 5/1 mm; field of view, 20 to 40 cm; matrix, 128 × 128; excitation, 2. A b-value of 0 and 1,000 s/mm^2^ were also applied in three orthogonal directions. Post-contrast-enhanced axial and sagittal T1-weighted imaging were also performed using the parameters described above with the exception of the 5 mm slice thickness.

#### MR and MR-DW image analysis

Conventional MR and DW-MR imaging data were analyzed on an Advantage Windows workstation 4.2 (GE Healthcare, Milwaukee, WI, USA). Image analysis was carried out by two radiologists in consensus (with 7 and 9 years’ experience in musculoskeletal MR imaging, respectively). The signal intensity of the solid components on T2-weighted MR images was defined as intermediate or high, relative to the muscle signal and signal intensity of the solid portions exhibiting enhancement post-injection. The signal intensity of the solid components at b = 1,000 s/mm^2^ on the DW images was defined as intermediate or low relative to that of the muscle.

#### Data calculation and analysis

The solid components of the lesions were identified on T2-weighted and post-contrast T1-weighted images, and were matched on ADC maps. The ADC values of the solid components in each tumor were measured on DW images by a radiologist using an Advantage Windows workstation 4.2 and FuncTool software (GE Medical Systems). In order to minimize variability, the largest possible region of interest (ROI) was placed manually in the solid part of the tumor in each image (range: 10 to 80 mm^2^). If the lesion exhibited irregular or heterogeneous solid components, two or three ROIs were drawn within the targeted components and the mean ADC value was calculated for the analyses.

#### Statistical analysis

All analyses were performed using SPSS v. 13.0 software for Windows (SPSS; Chicago, IL, USA). Differences in the mean ADC values of the bone tumors between the benign and malignant groups were evaluated using Student’s *t*-test. A value <0.05 was considered statistically significant. Biopsy or surgical pathology results were used as reference standards for assessment of the bone tumors. Receiver operating characteristic curve analysis was performed to assess the diagnostic performance of the mean ADC values in characterization of benign and malignant bone tumors.

## Results

### Demographics and histopathological characteristics

The histopathological types of 198 bone masses in the 178 patients are summarized in Table 1. The study group consisted of 81 males and 97 females, with a mean age of 31.52 ± 28.31 years (range: 1 to 92 years). These included 131 patients with solitary bone tumors, 22 patients with 28 Langerhans cell histiocytosis, and 25 patients (n = 15 lung cancer, n = 4 breast cancer, n = 3 prostate cancer, and n = 3 colorectal cancer) with 39 metastatic bone tumors. A total of 84/198 (42.4%) bone masses were benign and 114/198 (57.6%) were malignant. The diameters of the lesions in the benign group were 1.5 to 11.2 cm (median: 3.7 cm) and 1.2 to 16 cm (median, 4.1 cm) in the malignant group.

**Table 1:** **Histological type and apparent diffusion coefficient (ADC) values of 198 bone masses (mean ± SD × 10** ^**−3**^ **mm** ^**2**^ **/s)**

Type of bone masses	No. of lesions	High SI on T2WI (%)	High SI on DWI (%)	Range of ADC values	Mean ADC value
**Benign bone lesions**	84	54 (64.3)	50 (59.5)	0.49–1.59	1.17 ± 0.36
Non-ossifying fibroma	17	4 (23.5)	2 (11.8)	0.49–0.99	0.78 ± 0.17
Osteofibrous dysplasia	18	3 (16.7)	1 (5.6)	0.89–1.19	0.97 ± 0.17
Chondromyxoid fibroma	6	5 (83.3)	4 (66.7)	1.18–1.59	1.33 ± 0.15
Langerhans cell histiocytosis	28	28 (100)	28 (100)	0.96–1.55	1.29 ± 0.18
Giant cell tumor of bone	15	14 (93.3)	15 (100)	0.98–1.47	1.21 ± 0.20
**Malignant bone lesions**	114	101 (88.6)	105 (92.1)	0.58–1.35	0.86 ± 0.20
Chordoma	27	21 (77.8)	24 (88.9)	0.59–1.11	0.80 ± 0.14
Ewing sarcoma	9	6 (66.7)	7 (77.8)	0.70–0.91	0.82 ± 0.07
Osteosarcoma	8	6 (75.0)	5 (62.5)	0.87–1.02	0.97 ± 0.08
Chondrosarcoma	12	10 (83.0)	11 (91.7)	0.69–1.21	0.94 ± 0.15
Plasmacytoma	8	8 (100)	8 (100)	0.68–1.12	0.86 ± 0.15
Primary lymphoma	11	11 (100)	11 (100)	0.68–1.09	0.87 ± 0.14
Metastatic bone tumor	39	39 (100)	39 (100)	0.58–1.12	0.81 ± 0.14

SI, Signal intensity; T2WI, T2-weighted image; DWI, Diffusion-weighted image.

### Relationship between DW imaging and bone tumor types

The percentage of T2-weighted MR images of the solid components in bone masses giving intermediate or high signal intensities was significantly lower in the benign tumor group compared to the malignant tumor group (64.3% and 88.6%, respectively; *P* <0.01). The 84 benign lesions with homogeneous or heterogeneous low signal intensities included 76.5% non-ossifying fibromas, 83.3% osteofibrous dysplasia tumors, 16.7% chondromyxoid fibromas, and 6.7% giant cell tumors of bone.

**Figure 1:**
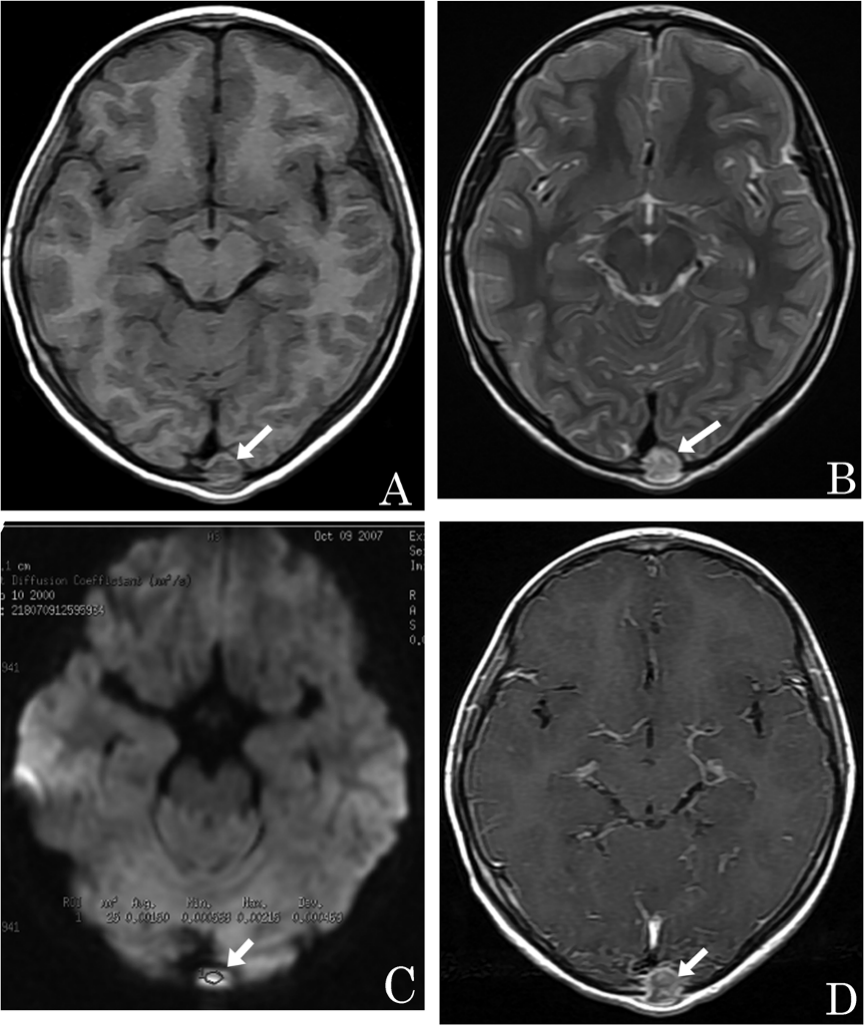
**A 7-year-old girl with an eosinophilic granuloma.** **(A)** Axial T1-weighted image showing an isointense small round mass within the occipital bone (arrow). **(B)** An axial T2-weighted image reveals that the mass is hyperintense (arrow). **(C)** Axial DW imaging reveals that the mass is slightly hyperintense with a high ADC value (circled area: ADC = 1.50 × 10^−3^ mm^2^/s) (arrow). **(D)** Axial enhanced T1-weighted image showing the lesion with marked enhancement (arrow).

**Figure 2:**
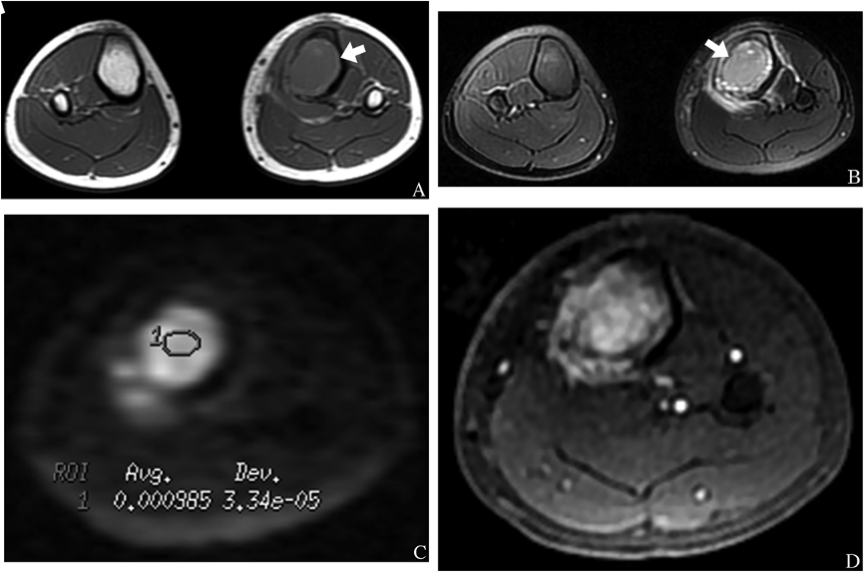
**A 23-year-old man with an osteosarcoma.** **(A)** An axial T1-weighted image showing a hypointense left tibial osteosarcoma mass (arrow). **(B)** Axial T2-weighted image showing that the mass has high signal intensity (arrow). **(C)** Axial DW imaging reveals that the mass is hyperintense with a low ADC value (circled area: ADC = 0.985 × 10^−3^ mm^2^/s). **(D)** Axial contrast-enhanced T1-weighted image shows marked enhancement of the bone mass.

Evaluation of the bone masses by DW imaging at b = 1,000 s/mm^2^, revealed that a significantly higher proportion of malignant lesions exhibited a high signal intensity within the solid components of the bone masses compared to those in benign lesions (92.1% and 59.5%, respectively; *P* <0.05). Further analysis of the images showed that the presence of a solid component with high signal intensity on T2-weighted images and high signal intensity on DW images with low ADC values (<1.10 × 10^−3^ mm^2^/s, at b = 1,000 s/mm^2^) could be considered predictive of malignancy (Figure 1). Conversely, the presence of a solid component with high or low signal intensity on T2-weighted images and low signal intensity on DW images with high ADC values (≥1.10 × 10^−3^ mm^2^/s, at b = 1,000 s/mm^2^), or low signal intensity on T2-weighted images and low signal intensity DW images with low ADC values (<1.10 × 10^−3^ mm^2^/s, at b = 1,000 s/mm^2^) could be considered predictive of a benign mass (Figure 2).

### ADC analysis

The mean ADC values of the solid components in the bone masses were determined for each group. There was considerable overlap in the range of values observed within the benign and malignant bone tumors; however, the mean ADC value for benign tumors (1.17 ± 0.36 × 10^−3^ mm^2^/s) was significantly higher than that in malignant tumors (0.87 ± 0.20 × 10^−3^ mm^2^/s; *P* <0.05). When the tumor subtypes were compared, no significant difference in the ADC values was found between the non-ossifying fibromas or osteofibrous dysplasia and malignant bone tumors (*P* >0.05). When an ADC cutoff value ≥1.10 × 10^−3^ mm^2^/s was applied, the benign and malignant bone tumors could be differentiated with a sensitivity of 89.7%, a specificity of 84.5%, a positive predictive value of 82.6%, and a negative predictive value of 95.3%, suggesting that this may be the optimal cutoff value for the ADC.

## Discussion

Our results demonstrated that the presence of a solid component in bone lesions with high signal intensity on DW and T2-weighted images, combined with low ADC values can be used to distinguish between malignant and benign bone lesions. These findings suggested that DW imaging with quantitative analysis of ADCs may improve the diagnostic performance of bone MR imaging, and provide clinically valuable information on the tumor microenvironment.

DW imaging has been extensively investigated for its ability to characterize tissue in various lesions. It has been shown to increase the accuracy in distinguishing benign from malignant masses, and discriminating between metastatic and benign lymphadenopathies. It has also proved successful in the evaluation of cerebral ischemia and intracranial tumors [1, 3, 4]. Malignant tumors with a high nuclear/cytoplasmic ratio, hypercellularity, and a reduced extracellular matrix often have restricted mobility of water molecules and low ADC values, whereas benign tumors generally have higher ADC values. These variations were reflected in our results. Although there was some overlap in the ADC values between the malignant and benign bone lesions, the mean ADC values in the 114 malignant bone tumors was significantly lower than that in the 84 benign bone masses. Our results further revealed that the optimal ADC cutoff value for differentiating between benign and malignant tumors was 1.10 × 10^−3^ mm^2^/s. This result was consistent with those given in previous reports [7–12].

A study by Hayashida et al. [4] evaluated the contribution of DW imaging in combination with quantitative analysis of ADCs in the characterization of 20 bone masses, including 8 solitary bone cysts, 5 fibrous dysplasia tumors, and 7 chondrosarcomas. Their results suggested that this method of imaging bone lesions was not suitable for differentiating between benign and malignant bone lesions. This apparent discrepancy may have been due to differences in pathological bone architecture. However, our results revealed a significant difference in the mean ADC values of the solid components between malignant and benign bone lesions. In addition, our results showed low ADC values in 17 non-ossifying fibromas and 18 osteofibrous dysplasia tumors. These may have been due to the presence of abundant collagen-producing fibroblastic cells and a dense network of collagen fibers within the extracellular matrix, which can restrict the Brownian motion of water molecules. This characteristic may also have been responsible for the absence of a significant difference between the mean ADC values of non-ossifying fibromas or osteofibrous dysplasia tumors and malignant bone lesions.

Our results showed that 88.6% of the T2-weighted images of solid components in malignant tumors displayed high signal intensities, compared to 11.4% which displayed low signal intensities. However, this value increased to 92.1% when the DW images with low ADC values were analyzed. Bone lesions which exhibited low signal intensity on T2-weighted images and high signal intensity on DW images may have resulted from solid components with increased nuclear/cytoplasmic ratios and hypercellularity due to a reduction in both the extracellular matrix and the diffusion space of water protons in the extracellular and intracellular dimensions. In addition, the low signal intensity on T2-weighted images and DW images of benign bone lesions, such as non-ossifying fibromas and osteofibrous dysplasia tumors, may have been due to the high density of fibers, low cellularity, and low water content in both the extracellular and intracellular spaces [4, 13]. Our findings confirmed that a high signal intensity on DW images of solid components with low ADC values can serve as a useful criterion for predicting malignancy in bone lesions, and that a low signal intensity on T2-weighted images and DW images of solid components with low ADC values may be an effective criterion for predicting the presence of benign disease.

Our study had the following limitations: DW imaging often has poor spatial resolution, therefore, drawing the ROI on DW images while viewing T2-weighted or contrasted T1-weighted images may result in information bias. A better approach may be to fuse the DW images of solid components that show an abnormal signal at b = 1,000 × 10^−3^ mm^2^/s onto structural images in order to accurately position the ROI. Furthermore, the proposed value for the ADC threshold will need to be validated in a larger group of patients, as the ADC can be affected by many factors, including magnetic susceptibility, spatial resolution, signal to noise ratio, and the pathophysiological characteristics of the bone lesions.

## Conclusions

DW imaging is a potentially valuable method for differentiating between benign and malignant bone tumors as it offers both high sensitivity and specificity. Bone masses with solid components that exhibit low signal intensity on T2-weighted and DW images and low ADC values are invariably benign.

## Abbreviations

ADC: Apparent diffusion coefficients
DW: Diffusion-weighted
MR: Magnetic resonance
ROI: Region of interest.
